# Soundwalk path affecting soundscape assessment in urban parks

**DOI:** 10.3389/fpsyg.2022.1096952

**Published:** 2023-01-09

**Authors:** Chutian Sun, Qi Meng, Da Yang, Yue Wu

**Affiliations:** ^1^Key Laboratory of Cold Region Urban and Rural Human Settlement Environment Science and Technology, Ministry of Industry and Information Technology, Harbin, China; ^2^School of Architecture, Harbin Institute of Technology, Harbin, China

**Keywords:** soundwalk path, perceived sounds, soundscape assessment, urban parks, soundscape design

## Abstract

**Introduction:**

Previous soundscape studies focused on sound perception and acoustic comfort in sampled sites and ignored the characteristics of the experiential process along the paths. Therefore, the effects of soundwalk paths on soundscape assessment should be explored.

**Methods:**

In this study, a typical urban park was selected as a case study. The differences, characteristics, and effects of soundwalk paths on the soundscapes were explored, based on a soundwalk survey and laboratory study.

**Results:**

The results are as follows: first, in the soundwalk, different soundwalk paths in the urban park had significant effects on the perceived extent of individual sound and soundscape assessment. Second, the soundscape assessment was consistent with the peak-end rule. In the laboratory experiments, the peak assessment of soundscape appeared at the end location of the soundwalk paths, it was critical to the overall soundscape assessment. Furthermore, in the soundwalk, the overall perceived extent of individual sound was strongly affected by the perceived extent of dominant sounds at the end location of the soundwalk paths. Third, in the soundwalk, the sound loudness contrast path (noise-quiet/quiet-noise) and sound source contrast path (natural-artificial sound/artificial-natural sound) were compared. In the noise-quiet path, the assessment of acoustic comfort was higher than that in the quiet-noise path, and the assessment of subjective loudness was lower than that in the quiet-noise path (*p* < 0.01). The subjective loudness of the artificial-natural sound path was lower than that in the natural-artificial sound path (*p* < 0.05).

**Discussion:**

Thus, the design of the soundwalk paths was expected to enhance visitors’ soundscape assessment of urban parks.

## 1. Introduction

A growing body of evidence suggests that the soundscape’s quality helps define the quality of the visitors’ experience of the park (Downing and Stusnick, 2000). Thus, numerous studies have investigated urban parks’ soundscapes, which, per prior findings, contribute to the improvement of environment’s quality, behavioral activities, and also impact landscape design (Annerstedt et al., 2013; Liu et al., 2013b; Yue et al., 2022). Payne (2013)—exploring the ‘restorative’ and ‘tranquil’ nature provided by soundscapes in typical locations of urban parks—found that soundscapes positively impact quality of life. Moreover, Van Renterghem et al. (2020) found enhancing natural-like sound sources improves users’ perceptual experience of parks’ environment through an experiment of one typical location in an urban park. Pérez-Martínez et al. (2018) reported that different proportions of sound sources exhibit different effects on users in different locations of city parks, and users’ sound preferences affect the perceived quality of the environment. Liu et al. (2018) conducted a survey in typical landscape nodes, demonstrated that behavioral factors, such as visit frequency and length of stay, related to the soundscape experience in urban parks. Yue et al. (2022) constructed an urban park design prediction model, which contains sound pressure level (SPL) prediction, sound source prediction, and soundscape assessment prediction, from a soundscape survey of characteristic nodes. The mentioned studies explored the soundscape’s role in sampled sites of urban parks. However, the continuous acoustic perception from a sequential perspective along the paths, which is an essential way to experience soundscape of urban parks, has received scant attention. Owing to the characteristics of users’ visiting behavior in urban parks, the experiential process along the paths—rather than the experience of sampled sites—is considered in this study.

In terms of urban parks’ paths, most studies have focused on the walking behaviors (Cohen et al., 2007; Kaczynski et al., 2008; Reed et al., 2008), physical activities (Kaczynski and Henderson, 2007; Kaczynski et al., 2008; Reed et al., 2008), micro-level design characteristics (Joseph and Zimring, 2007; Lu, 2010), destinations (Chudyk et al., 2015), and environmental comfort (Zhang and Dai, 2021). Actually, some soundscape studies have begun focusing on the effects of sound sequences, instead of soundwalk paths, on soundscape assessments. For instance, Västfjäll (2004) found that the recency effect—the phenomenon of sound remaining for a 1.5-min—affects an indoor space’s sound sequence evaluation. This effect was also observed by Aumond et al. (2017a,b) in a soundscape pleasantness assessment study which conducted in urban environments for very short walks. Nevertheless, this effect does not seem to be significant when longer routes are assessed (Aumond et al., 2017a). Based on a laboratory study, Wang et al. (2020) found that the perceptual extent of a specific sound source in a sound sequence would be reduced, compared to that of a simple sound source. This study predominantly focused on the effects of short-time sound sequences—or simple sound source—on sound sequence. In this study, the presentation-order effect has not been evidenced for most of the assessment locations. Therefore, the aforementioned studies cannot replace the long-time soundwalk path, which contains multiple sound source effects on sound sequences. Thus, how the soundwalk paths affect the soundscape in an actual urban park is unclear.

The peak-end rule has been applied to explain the characteristics of the experiential process. The peak-end rule was first proposed by Kahneman et al. (1993) in experiments on the ice water experience, which revealed the following rule in the process of experience: the results from the peak and end experiences effectively evaluate the quality of the whole experience process (Do et al., 2008). The peak-end rule has been observed in various research areas, such as aversive sounds (Schreiber and Kahneman, 2000), daily pain (Stone et al., 2000), interpretive programs at a cultural festival (Hwang and Choi, 2013), experiences of short vacation (Geng et al., 2013), gifts (Do et al., 2008), and affective evaluation and exercise behavior (Hargreaves and Stych, 2013). In the laboratory study of sound sequences, the peak-end rule effectively explains the interaction mechanism between the momentary and overall loudness assessment (Ponsot et al., 2013; Fiebig and Sottek, 2015). However, how the peak-end rule works in soundwalk paths of actual urban park is still unknown.

Soundscape evaluation quality (SEQ) is an essential indicator in urban park soundscape assessment. SEQ is evaluated from the aspects of sound pressure level (SPL), sound sources perception, and acoustic evaluation (Nilsson and Berglund, 2006; Brambilla et al., 2013). SPL invariably impacts (positively or negatively) soundscape assessment (Brambilla and Maffei, 2006; Nilsson and Berglund, 2006). Similarly, different sound sources also impact soundscape assessment (Liu et al., 2019). Positive sounds, such as natural sounds, are assumed to enhance acoustic comfort in urban parks (Tse et al., 2012; Ratcliffe et al., 2013; Liu et al., 2014a; Jeon and Hong, 2015). In terms of acoustic evaluation, acoustic comfort and subjective loudness are used in SEQ. Jeon et al. (2010) concluded that natural sounds—such as that of water—effectively enhance acoustic comfort. Meanwhile, Botteldooren et al. (1999) analyzed subjective loudness and sound pressure levels to identify and define the quiet space in urban parks. The aforementioned studies have examined the effects of loudness or dominant sound sources on SEQ. However, the relationship between special soundwalk paths—such as sound loudness changing paths or sound source changing paths—and SEQ has not been established in urban parks.

Therefore, this study aimed to explore the following three research questions:

1. Whether soundwalk paths can affect the sound source perception and assessment?
2. Whether the location of sound source perception and assessment can affect overall assessment of soundwalk paths?
3. Whether the loudness and types of sound sources in soundwalk paths can affect the sound perception and assessment?

This study administered a soundwalk survey and conducted a laboratory experiment to investigate the effects of soundwalk paths on soundscape in a typical urban park.

## 2. Methodology

### 2.1. Experimental design and procedure

**Figure 1:**
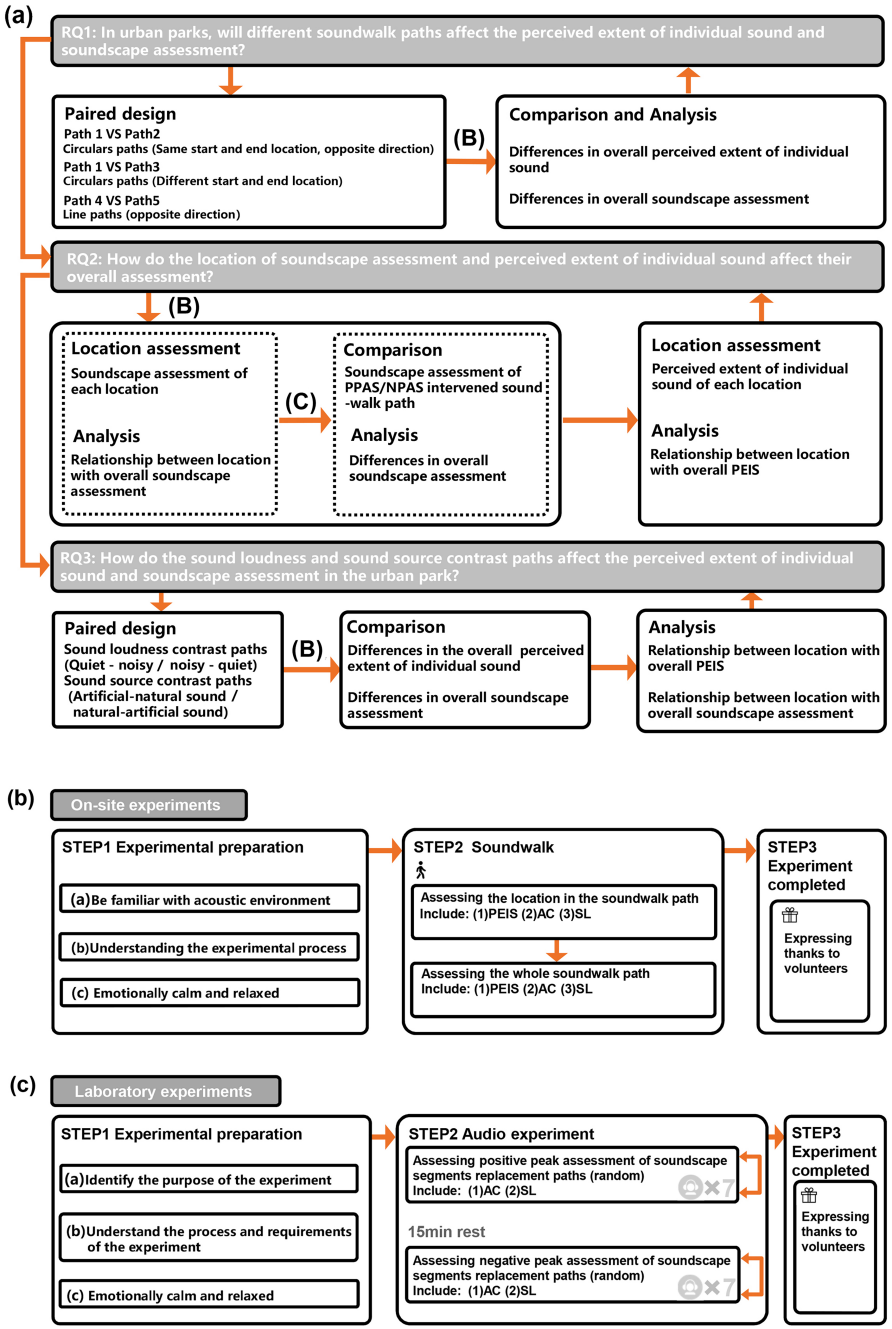
Experimental design and procedure for soundwalk and laboratory experiments: **(A)** Experimental design for the research question; **(B)** Procedure for soundwalk; **(C)** Procedure for laboratory experiments (PEIS, Perceived extent of individual sound; PPAS, Positive peak assessment of soundscape; NPAS, Negative peak assessment of soundscape; AC, Acoustic comfort; SL, Subjective loudness).

Figure 1 presents the integral experimental design and specific procedures for soundwalk and laboratory experiments. Figure 1A shows a comprehensive experimental design to investigate the three proposed research questions. The soundwalk and laboratory experiment steps are provided in Figures 1B,C, respectively. First, several paired paths were selected for the soundwalk to compare different paths’ effects on perceived extent of individual sound (PEIS) and soundscape assessment. Due to the location of the peak soundscape assessment, whether positive or negative, cannot be controlled in the soundwalk, the laboratory experiments are conducted to effectively control the location where the peak soundscape assessment appear. Thereafter, a combination of soundwalk and laboratory experiments of soundscape assessment, and PEIS in each location, were conducted to verify the peak-end rule’s applicability on the paths. Finally, the paired sound source and sound loudness contrast paths were selected for the soundwalk, to investigate the relationships between locations with overall PEIS and soundscape assessment.

### 2.2. Site survey

A typical urban park (Zhaolin Park) in Harbin, China, was selected as the site for the study (Sun et al., 2022). The park covers an area of 8.4 hectares and is divided into several areas by the park paths. The reasons for selecting the park are as follows: First, it contains different types of acoustics environments. The park contains quiet and noisy spaces, and natural and artificial sounds as the dominant sounds, which allow the investigation of the acoustics environment’s different characteristics. Second, it offers varied paths for visitors, thus providing a spatial contextual basis and various options for different sound environments.

**Figure 2:**
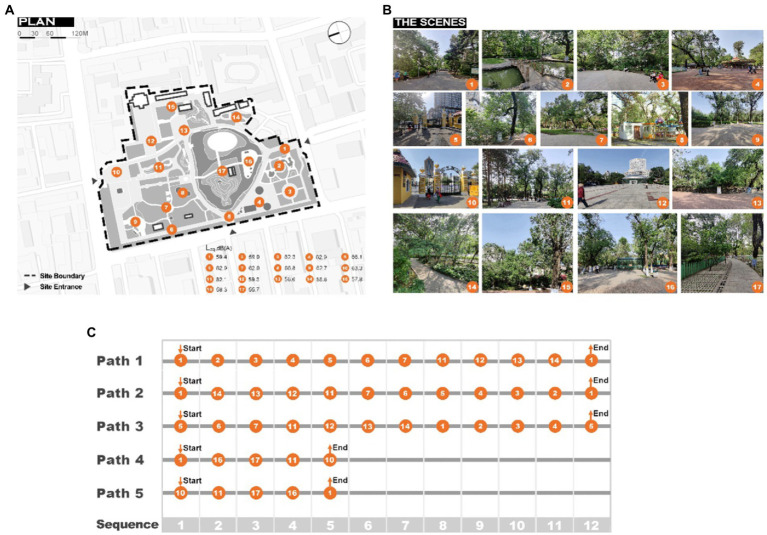
The basic information and the soundwalk paths **(A)** The plan of the survey site **(B)** Photos of locations in the park **(C)** The soundwalk paths.

The site survey contain a sound source and an SPL survey. The sound source questionnaire survey was based on the classification of sounds in urban public spaces (ISO/TS 12913–2:2018), and the sounds in the park were categorized into six main categories of sound as follows: human sounds, natural sounds, traffic noise, broadcasting sounds, music, and electro-mechanical sounds. The SPL in the park was assessed using equivalent continuous A-weighted sound pressure levels, measured by BSWA 801. The distance between the measurement location and a wall or other major reflective surfaces was at least 1 m, and the distance between the measurement location and the ground was 1.5 m (Zhang et al., 2016). The SPL was read, and instantaneous data were obtained—every second, for 5 min, in each measurement location. As presented in Figure 2, location 8 is the location with the highest SPL (Leq, 66.8 dBA), and location 17 is the location with the lowest SPL (Leq 55.7 dBA), whereas other locations exhibit SPLs of 57.8–66.1 dBA.

### 2.3. Soundwalk

In this study, the soundwalk was used to investigate the perceived sound and soundscape assessment of soundwalk paths. The soundwalk investigations were usually as a purposeful method of understanding the changing patterns of soundscape (Venot and Sémidor, 2006; Maffei, 2008; Jeon et al., 2011). However, the soundwalk are usually affected by complicated variables—such as seasons (Liisa et al., 2016), visual environment (Ren and Kang, 2015), crowd activity and density (Meng and Kang, 2016), temperature, humidity, wind speed, and illumination (Thwaites et al., 2005; Val et al., 2006; Meng et al., 2017, 2018). To reduce the effects of the aforementioned variables on the study, the following measures were adopted: the soundwalk was always limited to the same season’s period (Liu et al., 2013a; Jeon and Hong, 2015); thus, a time when the activity and density of crowd in the park tend to be consistent was chosen for the soundwalk (Meng and Kang, 2016).

According to the comparison of the visual landscape assessment results in the pre-experimental survey, the visual landscape environment of the established paths would not exhibit significant differences. Thus, the visual landscape environment variables’ effects could be controlled on the soundwalk. Considering the aforementioned variables, the soundwalk was conducted in rain-free, sunny weather, with temperatures in the range of 24–28°C, between 14:00–16:00 h, June–July 2021 (Meng et al., 2018; Zhang et al., 2019). Additionally, social factors also affected soundscape’s assessment (Deng et al., 2020). Therefore, in this study, the volunteers are university students of similar ages (min 20, max 28) were selected to participate in soundwalk (Liu et al., 2014b). Furthermore, the control of variables was also considered in the experimental design. Several visitors frequently take paths, which were considered carriers of soundwalk paths. Depending on the different paths’ characteristics, these paths were divided into three groups of two-paired samples for comparison and analysis. The paired path design counteracts the multifactorial variables’ effects in the soundwalk.

To investigate the effects of different soundwalk paths on the soundscape, two types of typical urban park soundwalk paths were selected: ‘sound loudness contrast paths’ (SL) and ‘sound source contrast paths’ (SS). SL was defined as the path leading from a quiet to noisy location (from Q to N), or vice versa (from N to Q). A subset of SL paired locations, Location 8 (noisy) and location 13 (quiet), were selected because of the differences in SPL, and artificial sounds are dominant in these locations. SS was defined as the path leading from the artificial to the natural sound location (from A to N), or vice versa (from N to A). A subset of SS paired locations, locations 12 (dominated by artificial sounds) and 15 (dominated by natural sounds), were selected because of the SPLs are almost the same, and do not interfere with the SL.

The field survey recruited 40 volunteers (mean age = 23.2, SD = 2.4), including 23 males and 17 females. All the volunteers had normal hearing according to their self-report. G-Power was used to analyze the minimum sample size of volunteers, the statistical test was chosen *a priori* two tailed Wilcoxon signed-rank test with matched pairs, assuming an effect size of *d* = 0.5, *α* = 0.05 and Power (1 − *β*) = 0.8 (Faul et al., 2009). The minimum average sample size required for the soundwalk was 35. The selected sample size was calculated using G-power software, and its validity was 85%, demonstrating that it fulfilled the requirements for statistical analysis.

Volunteers were randomly divided into groups (consists of 3–8 in a group) to avoid affecting the acoustic environment in the park. Each volunteer took a soundwalk at the pace of approximately 1.3–1.5 m/s. All volunteers were required to complete all 5 paths of the soundwalk, within the scheduled time, on the survey day. The experimental procedure presented in Figure 1B. To ensure that the volunteers could reflect on tis impact on their perception carefully, they were trained to be (a) familiar with the acoustic environment before the soundwalk, (b) familiar with the survey process, and (c) emotionally relaxed (Liu et al., 2014c). During the soundwalk, the perceived extent of individual sound, acoustic comfort, and subjective loudness were evaluated at the passing locations in each path. After completing each path, the overall perceived extent of individual sound, and the assessment of acoustic comfort and subjective loudness, were completed. A five-minute break was taken between each soundwalk path, to avoid interruptions between different paths.

A five-point scale was used to evaluate the perceived extent of individual sound categories, acoustic comfort, and subjective loudness, according to the survey method in ISO 12913-2 (ISO, 2018). Before the soundwalk, a detailed interpretation of the indicators was explained to the participants. The perceived extent of individual sound was assessed as ranging from ‘not at all’ (1) to ‘dominates completely’ (5). Due to the high degree of acoustic comfort was shown as comfortable, and the high degree of subjective loudness was shown as noisy. Acoustic comfort was assessed as ranging from ‘uncomfortable’ (1) to ‘comfortable’ (5). Subjective loudness was assessed as ranging from ‘quiet’ (1) to ‘noisy’ (5).

### 2.4. Laboratory experiments

Based on the results of the soundwalk, laboratory experiments were conducted, to further investigate the effect of the location where the peak soundscape assessment appeared, on the overall soundscape assessment.

For the typical soundscape fragments in the park, binaural soundscape clips were sampled, with the HEAD SQuadriga II recorder and BHS I headphone, based on the results of the soundscape assessment, for each location in the path during the soundwalk. Location 15 was selected as the sampling location for the positive-peak soundscape assessment, Location 8 for the negative-peak soundscape assessment, and Location 1 for the general soundscape assessment. ArtemiS SUITE 12.0 was used to edit the captured materials, namely, the positive (highest) or the negative (lowest) peak assessment of soundscape segments (PPAS/NPAS), and the general assessment of soundscape segment (GAS), into three types of clips, of 30 s each (Wang et al., 2020).

**Figure 3:**
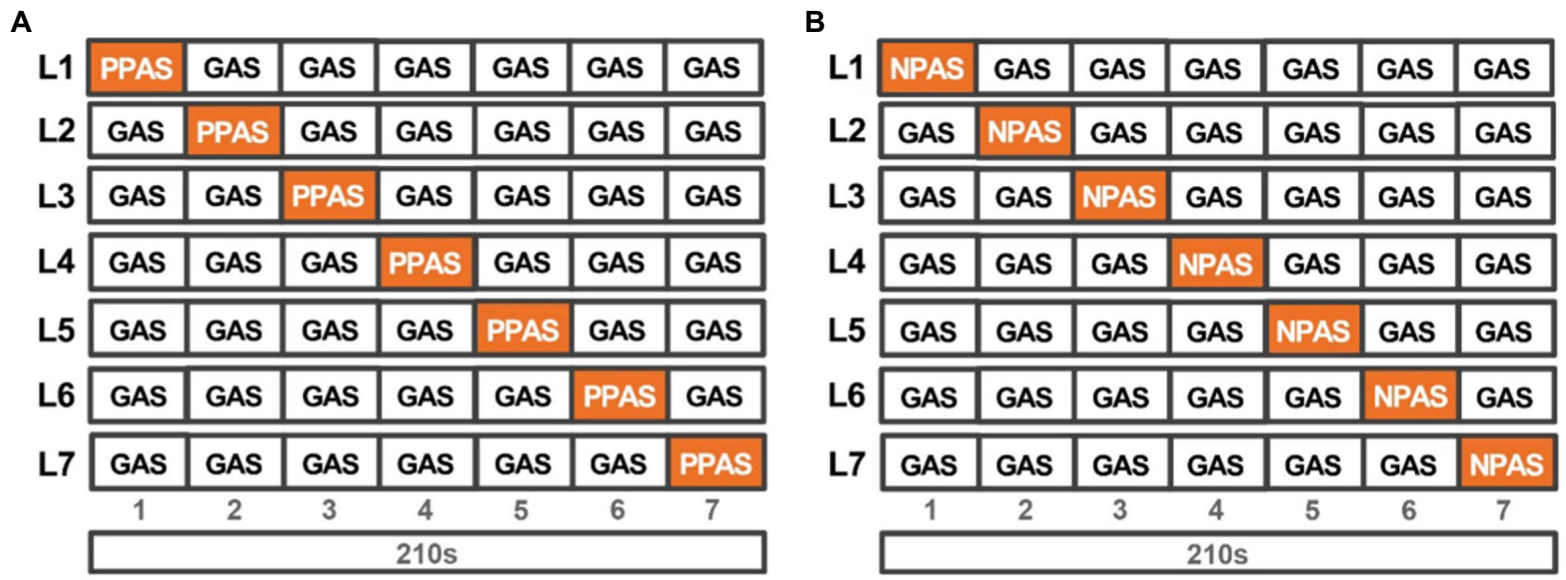
Composition of laboratory audiometric sound experience paths: **(A)** Composition of a PPAS-intervened laboratory audiometric sound experience paths; **(B)** Composition of a NPAS-intervened laboratory audiometric sound experience paths (PPAS, Positive peak assessment of soundscape segments, NPAS, Negative peak assessment of soundscape segments, GAS, general assessment of soundscape segment).

As presented in Figure 3, seven identical general experience segments, of 30 s each, were used as the basic segments, to avoid the segments containing a variety of assessment results, and simulate a typical 3–4 min soundwalk path. The PPAS/NPAS were substituted for the GAS at different locations, to create the paths of seven different locations of the PPAS and NPAS, and resulting in two groups of 14 soundwalk paths.

Based on previous studies, the usual number of volunteers for laboratory audiometric experiments should be more than 32 (Hongisto et al., 2016). Therefore, 45 volunteers were recruited to participate in this experiment (mean age = 23.5, SD = 2.5)—19 males and 26 females, all with normal hearing. We ensured that the volunteers could objectively reflect on the effects of the 14 paths on soundscape. Before the experiment, we ensured that (a) they clearly understood the purpose of the laboratory audiometry; (b) they were familiar with all the procedures and requirements of the experiment; and (c) they were calm and relaxed, so as not to interfere with the results of the assessment.

Each soundwalk path was re-played, using a HEAD SQuadriga II and a Sennheiser HD 660S. The experimental procedure is shown in Figure 1C, wherein the soundwalk paths are divided into two groups: the positive and negative peak assessment of soundscape segments intervention group. The two groups were tested randomly, with a 15-min break between groups (Wang et al., 2020). In each group, seven soundwalk paths were played in random order, with a 30 s interval between each path, and a final assessment of each path. Acoustic comfort was assessed on a scale ranging from ‘uncomfortable’ (1) to ‘comfortable’ (5), whereas subjective loudness was assessed on a scale ranging from ‘quiet’ (1) to ‘noisy’ (5).

### 2.5. Data analysis

The G-Power software was used to predict and determine the number of participants in the experiment. The results of the PEIS and the soundscape assessment were obtained using SPSS 24.0. The Shapiro–Wilk test was used to test the normality distribution of these results. The Wilcoxon signed-rank test was used to test the differences in the PEIS and the soundscape assessment in different soundwalk paths. The Friedman test was used to examine the effect of different locations of PPAS/NPAS in the soundwalk paths on the overall soundscape assessment. Spearman’s correlation analysis was used to analyze the correlation between each location of the PEIS and the overall PEIS in soundwalk paths, and the correlation between each location of the PEIS, soundscape assessment and the overall PEIS, soundscape assessment in the sound loudness and sound source contrast soundwalk paths.

## 3. Results

### 3.1. Different effects of soundwalk path on the soundscape assessment

**Figure 4:**
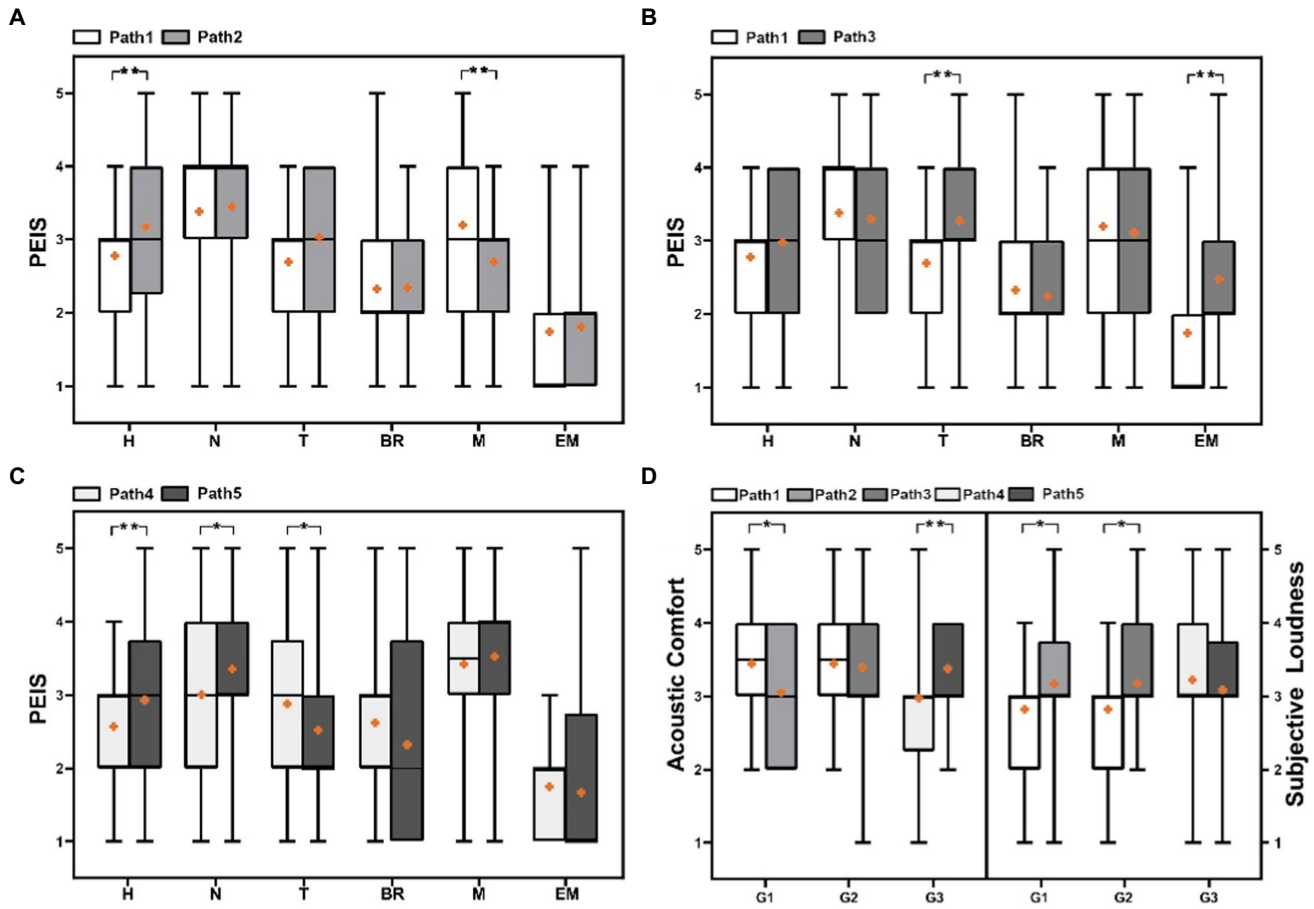
Comparison of the PEIS, acoustic comfort, and subjective loudness in different soundwalk paths: **(A)** PEIS in Path1 vs. Path 2, **(B)** PEIS in Path1 vs. Path 3, **(C)** PEIS in Path4 vs. Path 5, **(D)** Acoustic comfort and subjective loudness in G1, G2 and G3 (H, Human sound; N, Natural sound; T, Traffic noise; BR, Broadcast sound; M, Music; EM, Electro-mechanical sound. G1: Path 1 vs. Path 2, G2: Path 1 vs. Path 3 and G3: Path 4 vs. Path 5. *p < 0.05, **p < 0.01).

In this section, we divided the paths into 3 paired groups to compare the PEIS and soundscape assessment in different soundwalk paths, as shown in Figure 4. Figure 4A reveals that perceived extent of human sound and music has a significant difference (*p* < 0.01) in an opposite paired circle path (Path 1 and 2). Figure 4B shows that perceived extent of both traffic noise and electro-mechanical sounds has a significant difference (*p* < 0.01) in a synthetic paired circle path with different start and end locations (Path 1 and 3). Figure 4C indicates that the perceived extent of both human and natural sounds has a significant difference (*p* < 0.01 or *p* < 0.05) in an opposite paired line path (Path 4 and 5). These results shows that the perceived extent of some sounds have significant difference in different soundwalk paths.

Figure 4D shows that the subjective loudness and acoustic comfort also have significant differences in different paired soundwalk path groups. For instance, subjective loudness in Path 1 is significantly lower than that in Path 2 and Path 3 (*p* < 0.05), whereas the acoustic comfort in Path 1 is significantly higher than that in Path 2 (*p* < 0.05). The acoustic comfort is significantly higher in Path 5 than that in Path 4 (*p* < 0.01). These results indicates that the soundscape assessment also have significant difference in different soundwalk paths.

**Figure 5:**
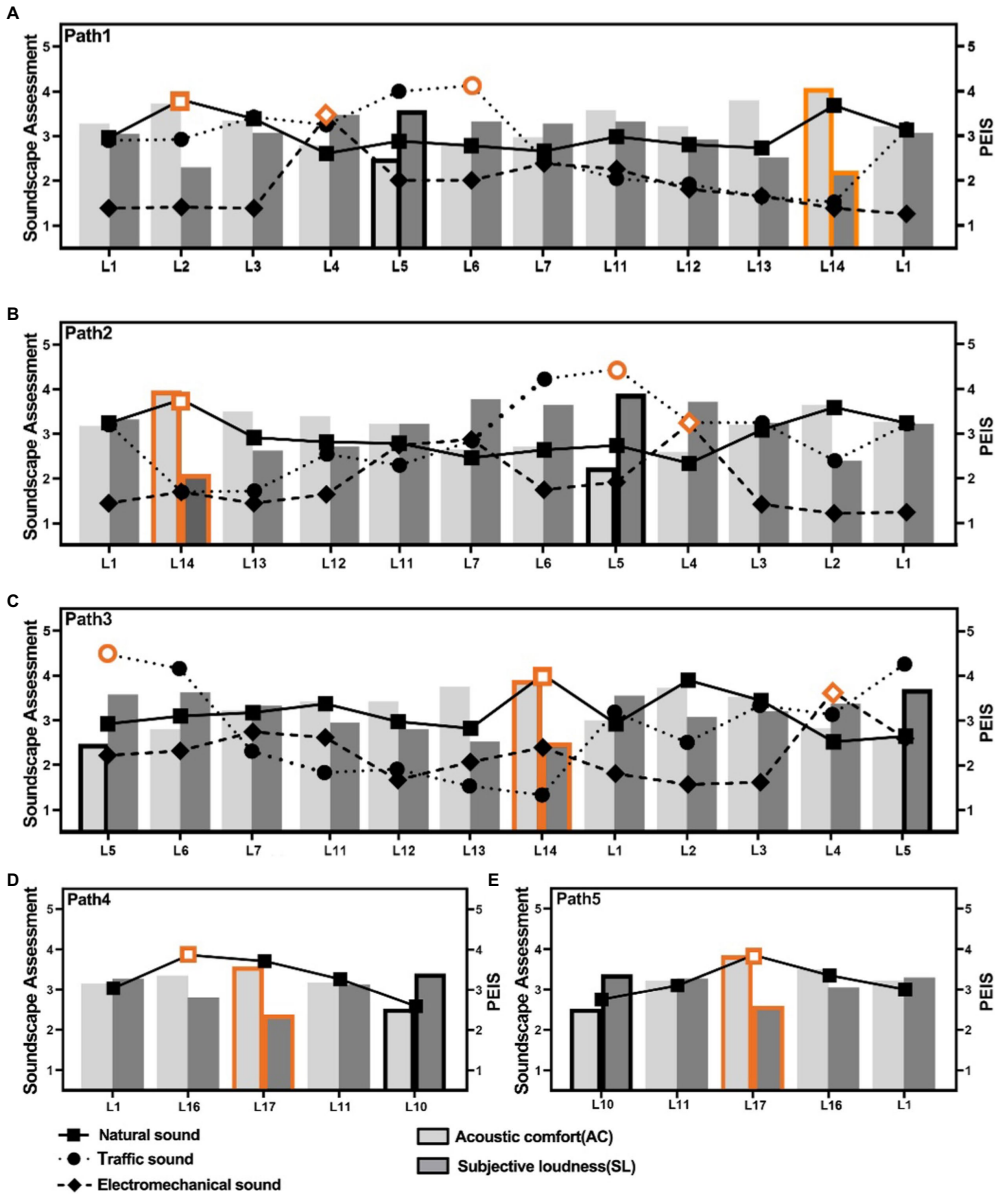
Soundscape assessment and PEIS at each location in the soundwalk paths: **(A)** soundscape assessment and the PEIS at each location in Path 1, **(B)** soundscape assessment and PEIS at each location in Path 2, **(C)** soundscape assessment and the PEIS at each location in Path 3, **(D)** soundscape assessment and the PEIS at each location in Path 4, and **(E)** soundscape assessment and the PEIS at each location in Path 5.

Figure 5 analyze whether the location of peak soundscape assessment and the location of the highest PEIS are the same in different soundwalk paths, as well as whether the locations of peak soundscape assessment and the highest PEIS can be changed in different soundwalk paths. The orange-colored dots in Figure 5 present the locations of the highest perceived extent of some sounds in the soundwalk paths, whereas the orange bars present the locations with the peak of the soundscape assessment.

Figure 5 shows that the location of peak soundscape assessment and the location of the highest PEIS are different. For instance, in the circle soundwalk paths of the case site, the peak of soundscape assessment at L14 while the location of the highest perceived extent of natural sounds at L2 in path 1. Besides, the highest or lowest peak of acoustic comfort will not be changed in different soundwalk paths. The highest PEIS will be changed in different soundwalk paths. For instance, in the circle line paths of the case site, the highest peak of soundscape assessment at L17 while the while the location of the highest perceived extent of natural sounds at L16 in path 4.

### 3.2. The effects of the location of soundscape assessment and PEIS on their overall evaluation

**Figure 6:**
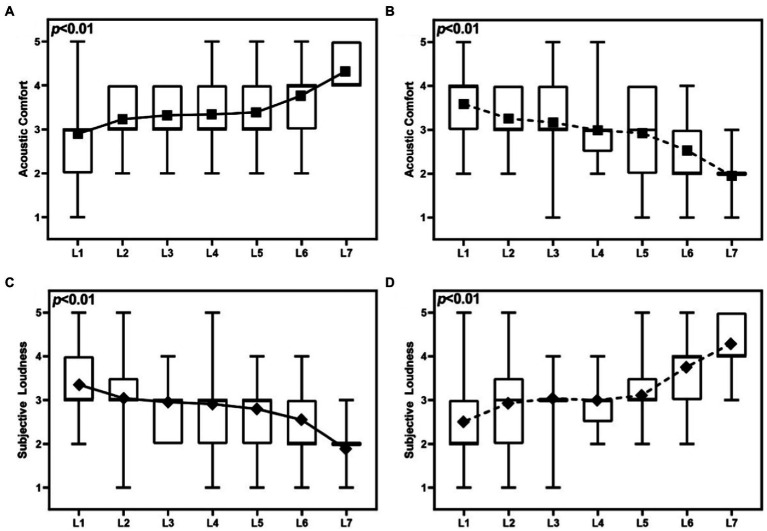
Results of different locations of peaks segments that affect acoustic comfort and subjective loudness in the soundwalk paths **(A)** Results of different locations of PPAS that affect acoustic comfort, **(B)** Results of different locations of NPAS that affect acoustic comfort, **(C)** Results of different locations of PPAS that affect subjective loudness, and **(D)** Results of different locations of NPAS that affect subjective loudness (L: The locations of assessment of soundscape segments appear in the soundwalk path).

To further investigate the effects of the peak soundscape assessment location on the overall soundscape assessment, the positive and negative peak assessment of soundscape segments was intervened in the same path, to explore the relationship between the location of the peak soundscape assessment and overall soundscape assessment (Figure 6).

Figure 6 presents how the different locations of PPAS/NPAS in the soundwalk paths exhibit significant effects on the overall soundscape assessment (*p* < 0.01). As presented in Figure 6A, with the PPAS moving toward the end of the path, an overall upward trend can be observed in acoustic comfort. Interestingly, the greatest increase is observed when the PPAS appears at Location 5 (near the end) to Location 7 (at the end), where the acoustic comfort increased, with a median from 3 to 4, and mean from 3.40 to 4.33. By contrast, as presented in Figure 6B, the acoustic comfort gradually decreases as the NPAS moves toward the end of the path. Interestingly, the fastest decrease was observed when the NPAS appeared at Locations 5 to 7, with acoustic comfort decreased, and a median from 3 to 2, and mean from 2.93 to 1.96.

As presented in Figure 6C, the subjective loudness gradually decreases as the PPAS moves toward the end of the path. Interestingly, the fastest decrease in the overall assessment of soundscape was noted when the PPAS appears at Locations 5 to 7, with subjective loudness decreased, and median from 3 to 2, and mean from 2.78 to 1.87. By contrast, as presented in Figure 6D, the subjective loudness gradually increases as the NPAS moves toward the end of the path. Interestingly, the greatest increase in subjective loudness is observed, when the NPAS appears at Locations 5 to 7, where the subjective loudness increased, and the median from 3 to 4, and mean from 3.09 to 4.27.

The above results suggest that the overall soundscape assessment changes sharply when the peak assessment segment appears near the end of the path as shown in Location 5 to 7. Thus, the closer the location, where peak soundscape assessment appears—whether positive or negative, to the end of the soundwalk path, the more critical effect on the overall soundscape assessment.

**Figure 7:**
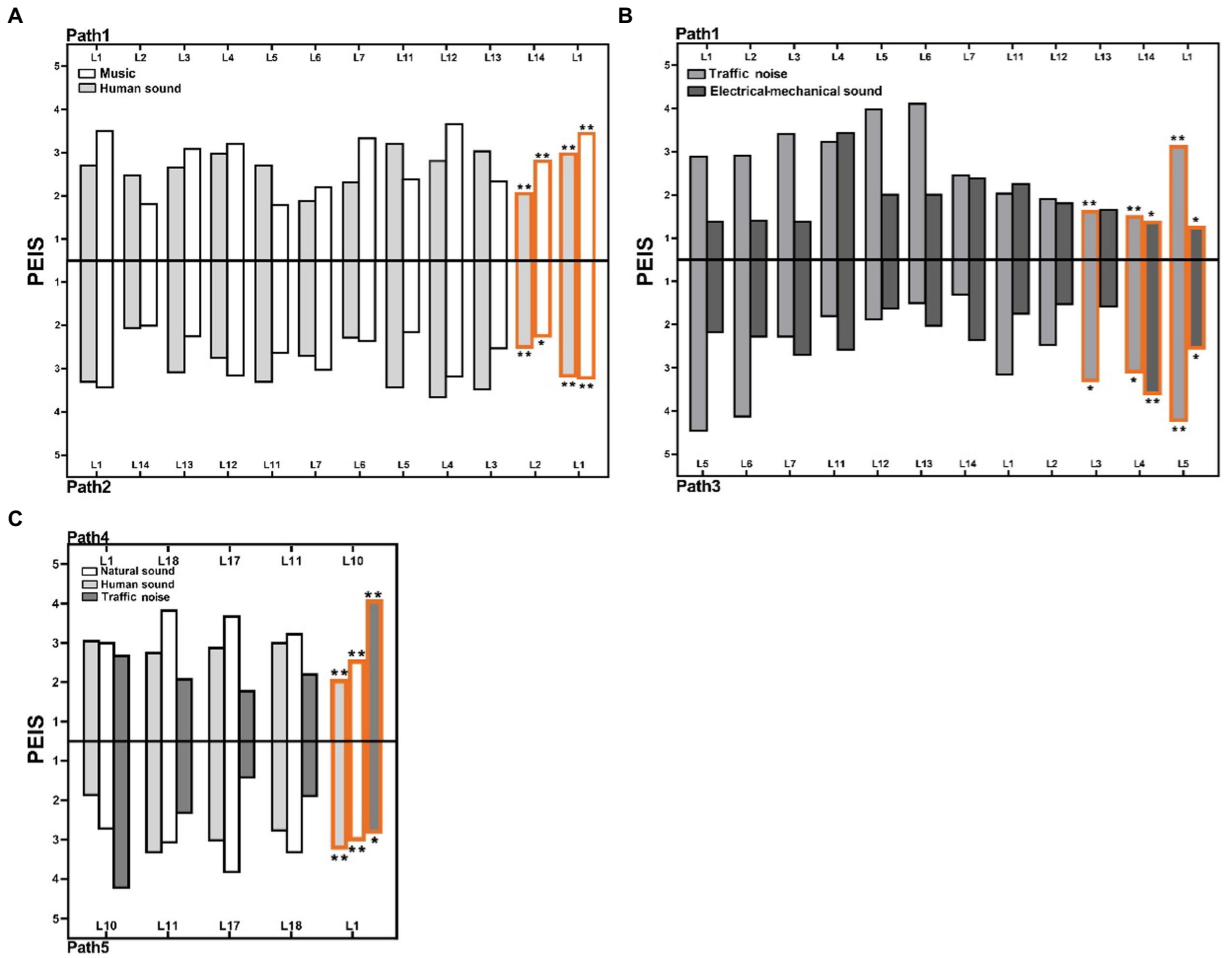
Comparison of PEIS, at each location, in paths with the differences in the overall PEIS: **(A)** Comparison of the perceived extent of human sound and music at each location in G1, **(B)** Comparison of perceived extent of traffic noise and electro-mechanical sounds at each location in G2, and **(C)** Comparison of perceived extent of traffic noise, human sound and natural sounds at each location in G3 (*p < 0.05, **p < 0.01: Correlation between perceived extent of sounds of each location, and the overall perceived extent of sounds in paths).

Figure 7 is to explore the relationship between the location of PEIS and overall PEIS. Figure 7A shows that the perceived extent of human sounds, at Location 14 and Location 1 in Path 1, is less than that at Location 2 and Location 1 in Path 2. This result explains why a significant difference in the overall perceived extent of human sounds was observed between the paths in Figure 4A. Similarly, in Paths 1 and 2, the difference in the perceived extent of music (Figure 4A) may be due to the perceived extent of music that is near the end and at the end locations (Figure 7A).

Figure 7B shows that the differences in the perceived extent of traffic noise and electro-mechanical sounds may be attributed to the perceived extent of these two sounds, near the end and at the end locations in the paths. This result explains significant difference exists in the perceived extent of traffic noise and electro-mechanical sounds, between Paths 1 and 3 in Figure 4B. Figure 7C shows that in Paths 4 and 5, the analysis of the differences in these sounds further illustrates that the differences in the overall perceived extent of sounds can also be attributed to the perceived extent of sounds at the end location. This result explains the perceived extent of human sounds, natural sounds, and traffic noise between Paths 4 and 5 in Figure 4C.

### 3.3. Effects of the sound loudness and sound source contrast paths on the soundscape assessment and PEIS

**Figure 8:**
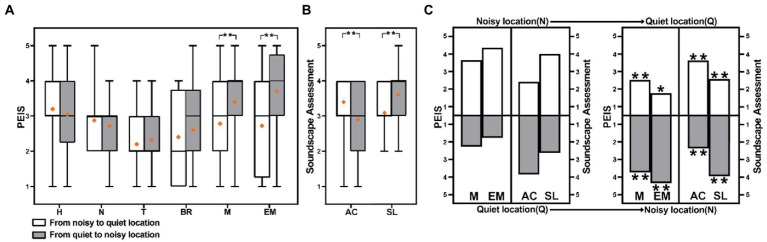
Comparison of PEIS and the soundscape assessment in sound loudness contrast paths **(A)** comparison of PEIS in sound loudness contrast paths, **(B)** comparison of the soundscape assessment in sound loudness contrast paths, and **(C)** comparison of each location PEIS and soundscape assessment in sound loudness contrast paths. (H, Human sound; N, Natural sound; T, Traffic noise; BR, Broadcast sound; M, Music; EM, Electro-mechanical sound; AC, Acoustic comfort; SL, Subjective loudness; *p < 0.05, **p < 0.01; **(C)**: *p, **p: correlation between PEIS of each location and overall PEIS, Assessment of each location and overall Assessment).

**Figure 9:**
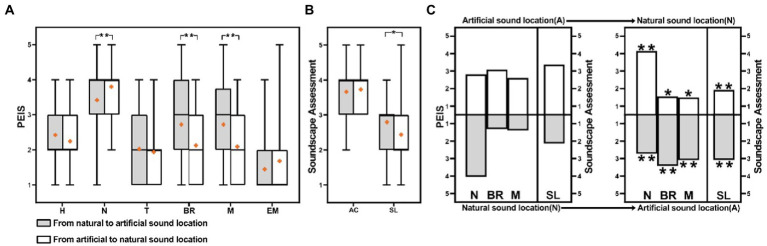
Comparison of PEIS and the soundscape assessment in sound source contrast paths: **(A)** Comparison of PEIS in sound source contrast paths, **(B)** Comparison of the soundscape assessment in sound source contrast paths, and **(C)** Comparison of each location PEIS and soundscape assessment in sound source contrast paths. (H, Human sound; N, Natural sound; T, Traffic noise; BR, Broadcast sound; M, Music; EM, Electro-mechanical sound; AC, Acoustic comfort; SL, Subjective loudness; *p < 0.05, **p < 0.01; **(C)**: *p, **p: correlation between PEIS of each location and overall PEIS, assessment of each location and overall assessment).

This section selected two typical contrast paths, named sound loudness (quiet-noisy and noise-quiet) and sound source (artificial-natural and natural-artificial sounds) contrast paths, to explore the effects on the perceived extent of sounds and soundscape assessment—through a comparative analysis as shown in Figures 8, 9.

Figure 8A reveals that the overall perceived extent of music is lower in the noisy-quiet path than in the quiet-noisy path (*p* < 0.01). The difference may be because the quiet location in the noisy-quiet path (2.525) exhibits a lower perceived extent of music than the noisy location in the quiet-noisy path (3.75). Similarly, the overall perceived extent of electro-mechanical sounds is lower in the noisy-quiet path than that in the quiet-noisy path (*p* < 0.01). This can be explained by the difference in the perceived extent of electro-mechanical sounds at the end location of the two paths, as presented in Figure 8C.

Figure 8B reveals that in the sound loudness contrast paths, the difference in acoustic comfort is significant (*p* < 0.01); although the median does not exhibit a difference, the mean difference is 0.5 (five-point scale). It demonstrates that the assessment of acoustic comfort is higher in the noisy-quiet path than that in the quiet-noise path. The difference in acoustic comfort may be attributed to the fact that the location at the end of the noisy-quiet path exhibits the highest—whereas the location at the end of the quiet-noisy path exhibits the lowest—acoustic comfort (Figure 8C).

Meanwhile, the difference in subjective loudness is also significant (*p* < 0.01). The subjective loudness of the noisy-quiet path is still lower than that of the quiet-noisy path; the median difference is 1.0, and the mean difference is 0.5 (five-points scale)—possibly because the quiet location at the end of the noisy-quiet path exhibits the lowest subjective loudness. By contrast, the noisy location at the end of the quiet-noisy path exhibits the highest subjective loudness, as presented in Figure 8C.

Figure 9A presents that the overall perceived extent of natural sounds, broadcast sounds, and music indicates a significant difference between the two paths (*p* < 0.01). Figure 9C presents that the difference may be that the perceived extent of natural sounds, at the natural location (4.15) in the artificial-natural sound path, is higher than that at the artificial location (2.725) in the natural-artificial path. Similarly, the overall perceived extent of music and broadcast sounds in the artificial-natural sound paths is lower than that in the natural-artificial paths (*p* < 0.01). The difference is attributable to the perceived extent of music and broadcast sounds, which appears at the end locations, in the two paths.

By analyzing the reasons for the significant difference in the perceived extent of music, natural, and broadcast sounds. The perceived extent of the sounds, at the end location in the sound source path, exhibits a significant effect on the overall perceived extent of sounds (Figure 9C).

A significant difference in the overall subjective loudness, between the two sound source contrast paths, is presented in Figure 9B. The subjective loudness of the artificial-natural sound path is lower than that of the natural-artificial sound path. The median difference is 1.0, and the mean difference is 0.35 (*p* < 0.05). The difference may be attributed to the fact that the subjective loudness of the end location, in the artificial-natural sound path, is the lowest, whereas the subjective loudness of the end location, in the natural-artificial sound path, is the highest (Figure 9C).

This section supports the results of Section 3.2, indicating that the soundscape assessment in the end location is critical to the overall soundscape assessment in the path. Accordingly, the end location, as the quiet location in the sound loudness contrast paths or the natural sound location in the sound source contrasts paths, results in a better soundscape assessment.

## 4. Discussion

The results in Sections 3.2 and 3.3 confirmed that the peak-end rule would affect soundscape experience assessment in the soundwalk path of urban parks. During a process of soundscape experience along soundwalk paths, the effect of the peak-end rule on soundscape perception is more often represented as one’s focus on the assessment of peak (positive or negative) and end locations. This effect is largely consistent with results on the momentary and overall sound source loudness assessment (Ponsot et al., 2013; Fiebig and Sottek, 2015), as has been shown in previous psychological research about the peak-end rule effect (Do et al., 2008; Geng et al., 2013; Hwang and Choi, 2013). In retrospective assessments, people always have a strong impression of the peak and the end experience in an experiential process (Fredrickson and Kahneman, 1993), especially when the peak experience appears as an end experience and influences the overall assessment. This provides an explanation for the soundscape perception in an experience process along the paths and applies to focus peak and the end experience in soundwalk paths on enhancing SEQ in urban parks.

Based on this, adjusting where the peaks soundscape assessment appeared in soundwalk paths further found that the peak soundscape assessments, whether positive or negative, appeared at the end location, which is critical to the overall soundscape assessment. This occurs because segments of soundscape experience need to be retrieved and aggregated over time for retrospective assessment of soundscape in soundwalk path, whereas it is not time-dependent or the number of soundscape experience segments (Tulving, 1984, 1993, 2002; Robinson and Clore, 2002a,b). Thus, people do not aggregate instances of particular soundscape experiences for retrospective assessment over a long time. This research selected positive or negative peak soundscape assessments to intervene in the path, especially when the peak soundscape assessment appears in the end location and the other soundscape assessments are ignored because of memory mechanisms (Ariely and Carmon, 2000). This is important to understanding how people make retrospective soundscape assessments of soundwalk paths in an urban park.

With the results of the study, we proposed three aspects that support the future additions to ISO 12913 concerning the perceptual assessment of soundscape quality in the following discussion.

### 4.1. Design strategies based on perceived sounds of the end location

In the soundwalk paths, the perceived extent of sounds at the end locations significantly affects the overall perceived extent of the sounds. The results provide a path perspective for sound source perception in urban parks and support the assessment of sound source perception in the ‘perceptual evaluation of soundscape quality.

Soundscape design usually focuses on the masking effectiveness of sounds, and the application of sound preferences. In terms of positive sounds, natural sound is usually preferred in urban parks. Previous studies have shown that natural sounds can effectively mask traffic noise, and improve noisy environments (Jeon et al., 2010; Van Renterghem et al., 2020). Therefore, natural sounds can be introduced near and at the end of the soundwalk paths, in urban parks. Previous study has revealed that people prefer natural sounds as they grow older (Yu and Kang, 2010). Therefore, locations are near and at the end of the soundwalk paths can be controlled, as a natural sound-dominated location, to create a type of soundwalk path that suits the sound preferences of aging people, and promotes good health.

In terms of negative sounds, traffic noise and electro-mechanical sound are usually disliked (Gramann, 1999; Ouis, 2001). Traffic sounds usually negatively affect the acoustics environment (Ouis, 2001; Booi and Van den Berg, 2012). Electro-mechanical sounds also negatively impact acoustics comfort and emotions. Therefore, it is essential to avoid, or reduce, the space of traffic noise and electro-mechanical sound, appearing at the end of the soundwalk path in urban parks.

The design of the perceived extent of sounds at the end of the soundwalk path, is recommended in urban parks. On the one hand, the masking effects of different sounds are used to enhance natural sounds, with a positive effect. For example, water sounds and birdsongs at the end of the soundwalk path can effectively mask more negative sounds. On the other hand, traffic noise or electro-mechanical sounds, should be avoided at the end locations of the soundwalk path. The aforementioned measures can be used to promote the quality of the soundscape in urban parks.

### 4.2. Effect of peak-end rule on soundscape optimization strategies

Studies of soundscape have interpreted the way to improve soundscape quality in different dimensions, including but not limited to noise control, sound masking, and soundscape subjective assessment, in urban parks. However, methods to control the quality of the soundscape in existing urban parks are not yet fully developed. The experimental results based on 3.2, not only provide a new perspective for improving SEQ in existing urban parks by controlling the location of the peak assessment of soundscape, at the end of the soundwalk path but also support the preparation of the ‘perceptual evaluation of soundscape quality’ for urban parks.

Landscape elements usually affect the assessment of the soundscape. Therefore, the design of elements can be used to modulate where the peak assessment of the soundscape appears, in the soundwalk path. The results of the presence of greenery and water features, contributed to a positive assessment of soundscape (Jeon et al., 2012). The presence of a water landscape, or an audio-visual environment containing a water landscape, impacts visitors’ experience (Liu et al., 2019). A high-quality visual environment reduces noise sensitivity and subjective loudness, and even improves noise tolerance (Samara and Tsitsoni, 2007). Meanwhile, the sound of water can effectively mask noise and, thus, contribute to the quality of the soundscape (Liu et al., 2019). Therefore, the end location of the soundwalk path, is where the water feature appears, in established urban parks. Alternatively, additional water features can be designed, at the end location of the soundwalk path, to enhance the existing soundscape quality.

It has also been reported that vegetation, plants, trees, and soil positively affect the quality of the soundscape (Fang and Ling, 2005). Generally, the A-weighted SPL is positively correlated with acoustic comfort (Yang and Kang, 2020), and vegetation can reduce it by reflecting, refracting, scattering, or absorbing sound, to promote acoustic comfort (Aylor, 1972; Fang and Ling, 2003). The effects of a vegetated sound barrier, on sound transmission, largely depended on the frequency of the sound (Yang et al., 2010), and the selection of plants constructed as barriers, with a noise reduction spectrum similar to the ambient noise spectrum. Thus, it can effectively suppress the transmission and effect of noise. In urban parks, green sound barriers can be added at the end of the soundwalk path, to enhance the soundscape quality. Additionally, a reasonable plant configuration can increase the quality of the soundscape. Yang et al. (2010) demonstrated that cedar reduces low-frequency noise more effectively than arrow-wood, oleander, or bamboo. Particular arrangements of vegetation can substantially attenuate a certain SPL of noise. Controlling the density, height, length, and width of vegetation is the most critical factor in reducing noise.

Therefore, water landscapes should be ideally placed at the end location, and design of vegetation should also be considered, for the soundwalk paths in urban parks.

### 4.3. Planning strategies for urban parks based on the sound loudness contrast paths and sound source contrast paths

In the planning stages of urban parks, the design of paths is usually dominated by visual landscape elements, and lacks consideration of the soundscape. The results of the experiment based on 3.3, provide a new reference for the pre-planning phase of urban parks, and gives the ‘perceptual evaluation of soundscape quality’ a new perspective for evaluation.

In the urban park, while comparing the two types of sound loudness contrast paths, acoustic comfort and subjective loudness of the quiet-noisy path, are better than that in the noisy-quiet path (*p* < 0.01). The above results confirm the results in section 3.2, and provide planning strategies to enhance the quality of the soundscape in urban parks. A noise-quiet path can be added to the urban park, to increase the assessment of the soundscape. Furthermore, based on the analysis and results of 3.3, quiet spaces should be placed at the end of the path. Based on the effect of the peak-end rule, this increases the quality of soundscape in the urban park.

When comparing sound source contrast paths, the subjective loudness of the artificial-natural sound path is lower than that of the natural-artificial sound path (*p* < 0.05). Therefore, in the stage of planning, the artificial - natural sound path can be added, to promote the assessment of subjective loudness in urban parks. In terms of acoustic comfort, no significant differences were found. The reason may be that changes in the dominant sounds do not cause significant differences in overall acoustic comfort, when the change in SPL is not significant. Thus, to maintain a lower SPL of the sound loudness path, increasing the artificial sound-natural sound path, can decrease the assessment of subjective loudness in urban parks. Furthermore, referring to the analysis and results of 3.2 and 3.3, the perceived extent of natural sounds tends to bring positive feelings (Medvedev et al., 2015), and the space in which the natural sound is the dominant sound should be placed at the end location of the path. Based on the effect of the peak-end rule, this improves the assessment of subjective loudness in the urban park.

### 4.4. Limitations and future prospects

In this study, the experiments of soundwalk are inevitably characterized by complicated factors. As highlighted in the methodology, the effect of multiple factors on soundwalk are reduced, by pre-experiments and experimental design.

Only one typical urban park was selected for this study. Although the Zhaolin Park is a representative urban park, it cannot represent all types of urban parks. In future studies, more attention should be paid to other types of urban parks. Moreover, the effects of only two different characteristics (sound loudness, sound source) soundwalk paths were investigated, on the soundscape in the urban park, whereas the soundwalk paths contain more than two different characteristic acoustics environments. Thus, different characteristics of soundwalk paths should be explored in future research.

Cultural factors and social contexts are usually two critical aspects of soundscape studies. There may be differences in soundscape assessment, based on different cultural and social contexts (Deng et al., 2020). Future studies should explore the effects of soundwalk paths on the soundscape of urban parks, in different cultural and social contexts.

Psycho-acoustic indicators—such as sharpness, roughness, and fluctuation—are generally used to interpret individual sound characteristics. This study focuses on how the paths affect soundscape assessment and the PEIS, from the sound experience perspective. Therefore, the psycho-acoustic characterization of individual sound in soundwalk paths will be explored in the following studies.

Further, the scope of experience should also include the assessment of soundscape and other contents, such as emotion or memory. Future research can further explore the effect of the soundwalk path, on these other dimensions.

## 5. Conclusion

This study investigated the effects of soundwalk paths on the soundscape of an urban park and provided the following conclusions.

In actual urban park scenes, which are influenced by soundwalk paths with the complex acoustic environments, the PEIS and soundscape assessment have a significant difference.

The soundscape assessment is consistent with the peak-end rule in the soundwalk paths of urban parks. The location where the peak assessment of soundscape appears at the end of the soundwalk path is critical to the overall soundscape assessment. Similarly, the overall PEIS is strongly affected by the perceived extent of dominant sounds at the end of the soundwalk path.

The relationship between soundwalk paths and SEQ in urban parks has been established. A comparison of the sound loudness contrast soundwalk paths and sound source contrast soundwalk paths, the PEIS of the end location has the greatest effect on the overall PEIS. Moreover, in the noise-quiet path, the assessment of acoustic comfort is higher than that in the quiet-noisy path. Meanwhile, the subjective loudness of the noisy-quiet path is lower than that of the quiet-noisy path. The subjective loudness of the artificial-natural sound path is lower than that of the natural-artificial sound path.

The study’s results support the future additions to ISO 12913 concerning the perceptual assessment of soundscape quality and provide new perspectives and ideas on how to assess and design soundscape for the company. More attention should be paid to the sound perception at the end locations of the soundwalk paths. In addition, designing landscape elements can modulate the peak assessment of the soundscape that appears at the end locations of the soundwalk path or increase the noise-quiet and artificial-natural sound paths during the planning stage.

## Data availability statement

The raw data supporting the conclusions of this article will be made available from the corresponding author upon reasonable request.

## Ethics statement

The studies involving human participants were reviewed and approved by Ethics Committee, School of Architecture, Harbin Institute of Technology. Written informed consent for participation was not required for this study in accordance with the national legislation and the institutional requirements.

## Author contributions

CS: methodology, visualization, writing – original draft, and data curation. QM: conceptualization, writing – review and editing, and funding acquisition. DY: investigation, data curation, and writing – review and editing. YW: methodology and investigation. All authors contributed to the article and approved the submitted version.

## Funding

This work was supported by the National Natural Science Foundation of China (NSFC) (grant numbers 52178070 and 51878210), Shanghai Key Laboratory of Urban Design and Urban Science, NYU Shanghai, Open Topic Grants (grant No. 2022QMeng_LOUD), and Natural Science Foundation of Heilongjiang Province (YQ2019E022).

## Conflict of interest

The authors declare that they have no known competing financial interests or personal relationships that could have appeared to influence the work reported in this paper.

## Publisher’s note

All claims expressed in this article are solely those of the authors and do not necessarily represent those of their affiliated organizations, or those of the publisher, the editors and the reviewers. Any product that may be evaluated in this article, or claim that may be made by its manufacturer, is not guaranteed or endorsed by the publisher.

## Abbreviations

GAS: general assessment of soundscape segment
NPAS: negative peak assessment of soundscape segments
PPAS: positive peak assessment of soundscape segments
SL: sound loudness contrast paths
SS: sound source contrast paths
PEIS: perceived extent of individual sound
